# The intracellular symbiont *Wolbachia* alters *Drosophila* development and metabolism **to** buffer **against** nutritional stress

**DOI:** 10.1371/journal.pgen.1011905

**Published:** 2025-10-15

**Authors:** Amelia R. I. Lindsey, Jason M. Tennessen, Michael A. Gelaw, Megan W. Jones, Audrey J. Parish, Irene L. G. Newton, Travis Nemkov, Angelo D’Alessandro, Madhulika Rai, Nicole Stark

**Affiliations:** 1 Department of Entomology, University of Minnesota, St. Paul, Minnesota, United States of America; 2 Department of Biology, Indiana University, Bloomington, Indiana, United States of America; 3 Department of Biochemistry and Molecular Genetics, University of Colorado Anschutz Medical Campus, Aurora, Colorado, United States of America; University of Pennsylvania, UNITED STATES OF AMERICA

## Abstract

The intracellular bacterium *Wolbachia* is a common symbiont of many arthropods and nematodes, well studied for its impacts on host reproductive biology. However, its broad success as a vertically transmitted infection cannot be attributed to manipulations of host reproduction alone. Using the *Drosophila melanogaster* model and natively associated *Wolbachia* strain “*w*Mel”*,* we show that *Wolbachia* infection supports fly development and buffers against nutritional stress. *Wolbachia* infection across two fly genotypes and a range of nutrient conditions resulted in reduced pupal mortality, increased adult emergence, and larger size. In parallel, transcriptomic and metabolomic analyses indicated that *Wolbachia* impacts a wide range of developmental and metabolic processes. *Wolbachia*-infected larvae had strong signatures of shifts in glutathione and mitochondrial metabolism, plus significant changes in the expression of key developmental regulators including *Notch*, the insulin receptor (*lnR*), and the juvenile hormone receptor *Methoprene-tolerant* (*Met*). We propose that *Wolbachia* can enhance host fitness by supporting fly development, especially during periods of nutrient stress.

## Author summary

*Wolbachia* is a bacterial symbiont of arthropods and nematodes, well described for its manipulations of arthropod reproduction. However, many have theorized there must be more to this symbiosis, even in well-studied *Wolbachia-*host relationships such as with *Drosophila*. Reproductive impacts alone cannot explain the success and ubiquity of this bacterium. Here, we use *Drosophila melanogaster* and their native *Wolbachia* infections to show that *Wolbachia* supports fly development and significantly buffers flies against nutritional stress. These developmental advantages might help explain the ubiquity of *Wolbachia* infections.

## Introduction

Maternally transmitted microbes have evolved numerous ways to alter host biology, ultimately facilitating their own transmission. Strategies include supplementation of the host’s diet via nutrient provisioning, protection against parasites and infections, and even direct manipulation of host reproduction [1]. Many insects, other terrestrial arthropods, and some nematode species are infected with bacteria in the genus *Wolbachia* (Alphaproteobacteria: Rickettsiales), a maternally transmitted intracellular infection long appreciated for its direct impacts on host reproduction and sex ratios [1–10]. Critically, *Wolbachia* can spread through populations in the absence of reproductive manipulations such as sperm-egg incompatibilities or sex ratio distortion [11]. And, increasingly, other *Wolbachia*-mediated benefits to the host are being uncovered [12].

For example, various *Wolbachia* strains can protect host insects against secondary infections, especially viruses, a phenomenon that now forms the basis of many ongoing vector control programs across the world [13,14]. Certain strains of *Wolbachia* are obligate beneficial nutritional symbionts, such as the bed bug-infecting *Wolbachia* that produce B-vitamins to support their obligately hematophagous (i.e., blood-feeding) hosts [15]. In other insects, facultative associations with *Wolbachia* have likewise been implicated in the balance of cholesterol, iron, or B-vitamins [16–19]. In *Drosophila*, there are increasingly examples of *Wolbachia* strains that have spread through populations without substantial reproductive manipulation of the host, implicating the importance of other fitness benefits [20,21]. Others have proposed that impacts on host metabolism and or *Wolbachia*-mediated pathogen protection may underlie such advantages [12,16,22–25]. In line with this, we previously identified that *Wolbachia* and virus interactively result in a suite of changes to the expression of adult *Drosophila melanogaster* nucleotide metabolism pathways [26].

While there are increasing examples in the literature that hint at significant metabolic interplay between *Wolbachia* and hosts, [16–19,26], our understanding of this symbiosis is still largely focused on reproductive processes and other aspects of adult biology [1]. Indeed, we know very little about *Wolbachia*’s interactions with hosts across development. In insects, not only is the juvenile period physiologically distinct (especially in holometabolous insects that undergo complete metamorphosis) but also this developmental period determines many aspects of adult biology and fitness [27–29]. A select number of studies have identified how *Wolbachia*’s physiology shifts across host developmental stages [30,31], and indeed it is clear that *Wolbachia* gene expression patterns are dynamic and significantly correlate with major shifts in host development. However, it remains to be seen if and how the hosts are impacted by the presence of *Wolbachia* during developmental. To address this significant gap in knowledge, we undertook a series of experiments using the fruit fly, *Drosophila melanogaster*, naturally infected with the *Wolbachia* strain *w*Mel. We hypothesized that *Wolbachia* plays a supporting role in host nutrition, and used a combination of transcriptomics, metabolomics, and diet manipulations, to investigate the relationship between developing flies and their *Wolbachia* infections.

## Results

### *Wolbachia* infection drastically alters larval gene expression

Given that the *Wolbachia*-host relationship is relatively unexplored in developing insects, we used a transcriptomics approach to broadly investigate the differences in larval biology when flies are infected with *Wolbachia*. We used an isogenic wild-type stock (DGRP-320) to assay gene expression in *Wolbachia* infected and uninfected flies at the second instar (L2) stage. For each library, we generated 28.6-38.4 million paired-end reads derived from A-tailed transcripts, of which 94.1-94.7% mapped to the *Drosophila melanogaster* reference (S1 Table, Fig A in S1 Text). We found that 1,477 genes were significantly differentially expressed due to the presence of *Wolbachia* (“DEGs”; 786 upregulated; 691 downregulated in the presence of *Wolbachia*; see Figs A and B in S1 Text, S2 and S3 Tables). DEGs had as much as ~100-fold change in expression. Included in the set of DEGs were two critical regulators of development, both downregulated in the presence of *Wolbachia*: *notch* and the insulin receptor *lnR.* Additional related DEGs included *lst* (a peptide hormone that suppresses insulin production, upregulated), *apolipophorin* (downregulated), *smr* (mediates repression of ecdysone and Notch signaling, downregulated), *ecdysis triggering hormone* and its receptor (*eth* and *ethr*, both upregulated), and the juvenile hormone (JH) receptor *met* (upregulated), plus a suite of JH binding proteins (*jhbp4*, -11, -12, and -16, all upregulated except for *jhbp11*).

**Fig 1:**
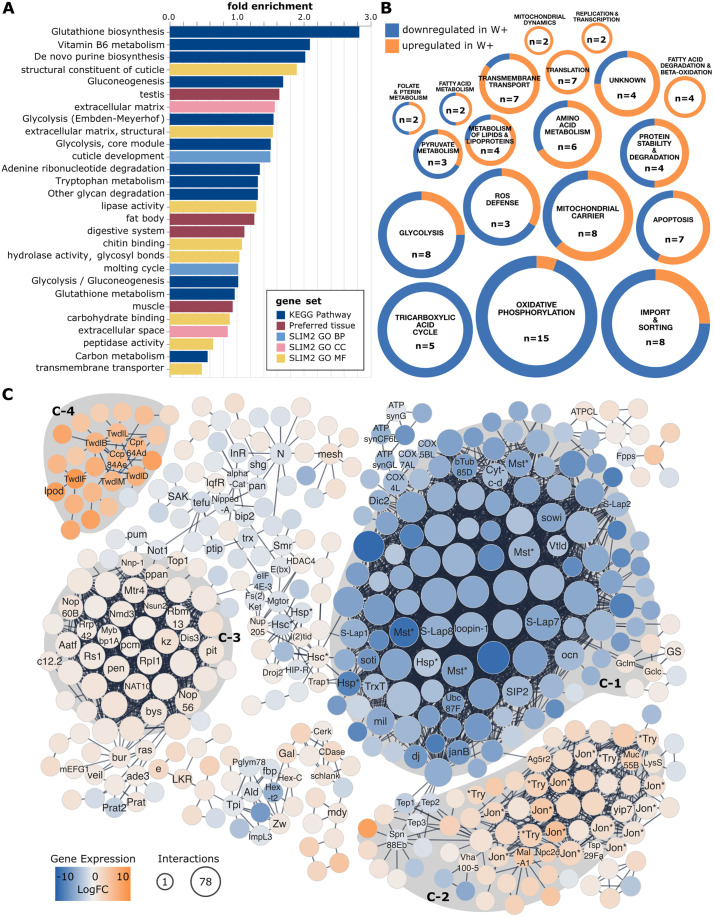
*Wolbachia* infection is associated with differential expression of 14% of the larval transcriptome. **(A)** Significantly enriched gene sets within the DEGs were assessed for enrichment using PANGEA [33]. Fold enrichment (log2) values indicate the amount of enrichment for a given gene set, and color coding corresponds to the database of origin for a given gene set. Gene Ontology (GO) gene sets are the *Drosophila* GO Subsets (GO slim) for Biological Process (BP), Cellular Component (CC), and Molecular Function (MF). **(B)** Mitochondrial related DEGs. Each circle represents a functional group, as defined by mitoXplorer, with colored slices indicating the proportion of genes in that group that were up- or down-regulated in response to *Wolbachia* infection. The values “n=” in each circle indicate the number of DEGs in that group, and the size of the circle is a relative indicator of the level of overrepresentation as compared to genomic background. **(C)** Core protein-protein interaction networks within the differentially expressed gene set. Nodes (proteins) are colored according to their change in gene expression relative to uninfected larvae. The size of each node corresponds to the number of interactions that protein has with others in the network. Nodes are labeled if the gene has a name, and either (1) has greater than two connections, or, (2) if the gene was discussed elsewhere in the main text. Some gene names are abbreviated using an asterisk to denote variations on the base family gene name. These include Jon* (e.g., Jon99Fii, Jon66 Ci, etc), Mst*, Hsp*, Hsc*, *Try (e.g., alphaTry, lambdaTry, etc). The full network with all nodes annotated with their full gene names is available as Fig B in S1 Text. Shaded regions denote clusters of the gene expression network that are significantly overrepresented with the following STRING terms: **(C-1)** uncharacterized peptidases, transmembrane transport, protein localization to microtubule, **(C-2)** signal, serine proteases, extracellular, hydrolase, digestion, carboxypeptidase, transmembrane transport, **(C-3)** ribosome biogenesis and tRNA modification, and **(C-4)** signal, cuticle development, chitin. Full details of all categories and statistical tests for the data represented in panels A and B are in S4 and S5 Tables, respectively.

Many genes involved in mitochondrial biology were also significantly differentially expressed (Fig 1). These included several mitochondrial ATP synthases (all downregulated), mitochondrial replication, transcription, and translation related proteins (e.g., mtDNA-helicase, MTPAP, mEFG1, mRpS10, LeuRS-m; all upregulated), heat shock proteins, transporters, and a suite of other enzymes involved in various aspects of mitochondrial metabolism (e.g., NADH dehydrogenase ND-20L, AIF, Gpo1, Sardh, mt:ND6, Acly, Taz, Tpi, amongst others). Importantly, the DEGs included several genes encoding components of the electron transport chain (Cyt-c-d, COX4L, COX5BL, COX7AL; all between 9- to 12-fold downregulated in *Wolbachia* infected larvae). Perhaps functionally related to the impacts on mitochondrial physiology, glutathione metabolism was largely upregulated in the *Wolbachia* infected flies (e.g., *gss1*, *gss2*, *gstE6*, *gstE9*, *gclm*, *gclc*, *gclm*), consistent with a number of previous reports that *Wolbachia* alters redox balance [13].

Notably, we also identified significant impacts of *Wolbachia* infection on the Toll and IMD immune pathways in the presence of *Wolbachia*. This includes downregulation of the Toll activator *spatzle* and its processing enzyme (SPE); downregulation of antimicrobial proteins including *attacins* (A, B, and D), *defensin*, and *baramicin-A1*; and differential expression of two pattern recognition receptors: peptidoglycan recognition proteins *pgrp-sa* (downregulated) and *pgrp-lb* (upregulated). We also saw a significant overrepresentation of unnamed genes: 36% of all the expressed genes were unnamed (e.g., only have the CG designation), whereas 47% of the DEGs were unnamed (X-squared = 69.12, df = 1, p < 0.0001).

To provide a more holistic look at the list of DEGs, we used two approaches: (1) over-representation analyses, and (2) network visualization based on protein-protein interactions. The DEGs were significantly overrepresented for 29 different gene sets including 13 KEGG pathways, 8 molecular functions, 4 preferred tissues, 2 biological processes (cuticle and molting), and 2 cellular components (both extracellular) (S4 Table, Fig 1A). Many overrepresented KEGG pathways have connections to mitochondrial biology and carbohydrate metabolism (e.g., glutathione biosynthesis/metabolism, glycolysis, gluconeogenesis, carbon metabolism, glycan degradation). Additionally, overrepresented KEGG pathways include vitamin B6 metabolism, *de novo* purine biosynthesis, adenine degradation, and tryptophan metabolism. Other overrepresented gene sets largely point towards digestion and nutrient storage. For example, preferred tissues included the digestive system, fat body (the primary site of fat storage), and muscle (the primary site of carbohydrate storage), and many overrepresented molecular functions were enzymatic (e.g., hydrolases, peptidases, lipases).

Given these signatures of altered mitochondrial biology, we specifically extracted mitochondrial processes from the DEG set and assessed expression across different functions (Fig 1B). A wide range of mitochondrial processes were represented in the DEG set. The most abundant DEGs, both in absolute number and degree of over-representation, were those related to oxidative phosphorylation: largely downregulated. Energy generation processes such as glycolysis and the tricarboxylic acid (TCA) cycle were similarly overrepresented and also largely downregulated. In contrast, transport-related functions (e.g., transmembrane transport and carriers) and core mitochondrial processes such as translation, transcription and replication, amino acid and protein metabolism, and mitochondrial dynamics were primarily upregulated. These data point to two contrasting impacts of *Wolbachia* infection on mitochondrial function: an up regulation of core processes but a down regulation of carbon and energy metabolism.

**Fig 2:**
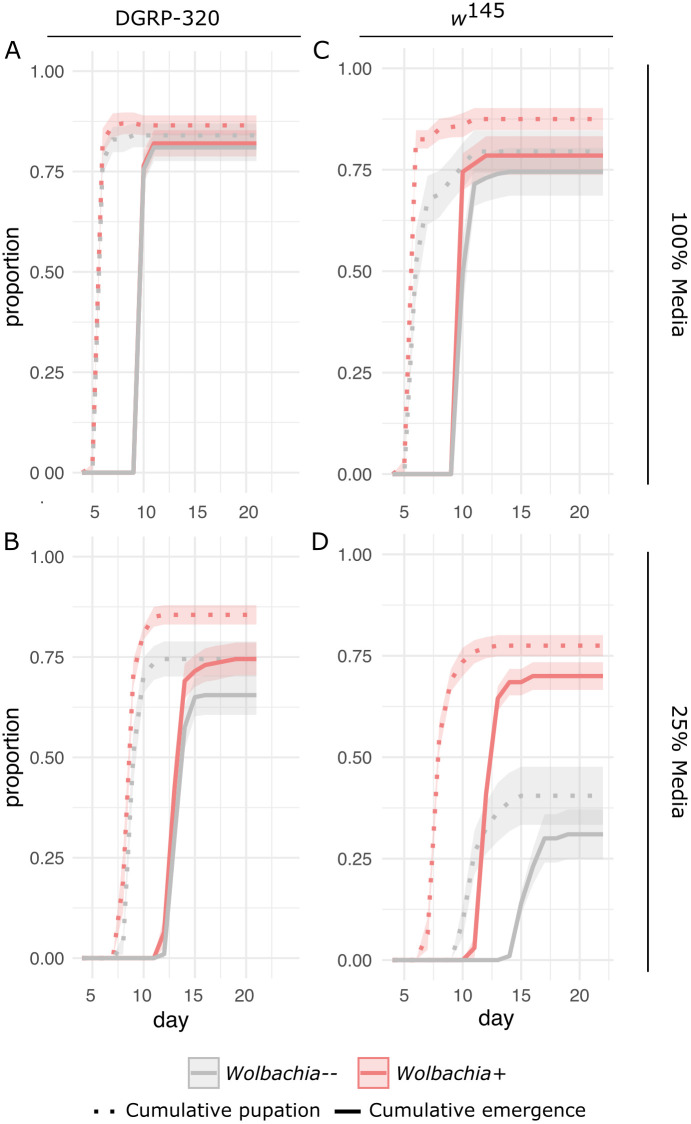
*Wolbachia* infection is beneficial under nutrient limited conditions. Flies (wild-type DGRP-320 and *w*^145^, with and without *Wolbachia*) were reared on different concentrations of media to test the impact of *Wolbachia* infection. Biological replicates included 20 larvae per each of 10 vials. Dotted and solid lines indicate cumulative pupation and adult emergence, respectively, with shaded regions defining standard error. All *Wolbachia*-infected treatments are in red, and *Wolbachia*-uninfected in grey. **(A)** Wild-type flies on 100%, and **(B)** 25% strength media. **(C)** *w*^145^ flies on 100% and **(D)** 25% strength media.

**Fig 3:**
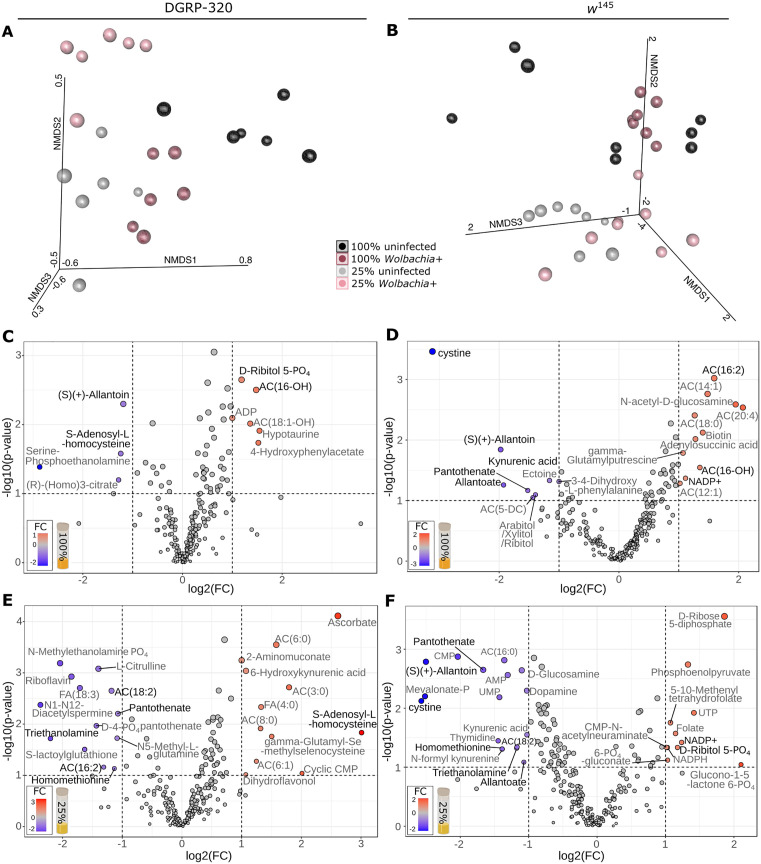
*Wolbachia* mediated impacts on the metabolome depend on host genotype and nutrition. **(A-B)** Three-dimensional non-metric multidimensional scaling (NMDS) plots indicating relative similarity of samples within the **(A)** DGRP-320 and (B) *w*^145^ genotypes. Point size indicates distance from the page, where larger points are closer. **(C-F)** Volcano plots indicating the relative abundance of specific metabolites in *Wolbachia* infected larvae relative to uninfected larvae: **(C)** DGRP-320 L2 larvae reared on 100% media. **(D)** *w*^145^ L2 larvae reared on 100% media. **(E)** DGRP-320 L2 larvae reared on 25% media. **(F)** *w*^145^ L2 larvae reared on 25% media. Metabolites with >2 log_2_ fold change (log2FC) and p < 0.1 are indicated, and, for those that met these criteria in >2 conditions (e.g., panels) the metabolite name is in black font rather than grey. “Phosphate” is abbreviated with “PO_4_”. “AC(#:#)” or “FA(#:#)” refers to acylcarnitines or fatty acids where numbers in parentheses indicate the total number of carbons in the chain, and the number of unsaturated bonds, respectively.

**Fig 4:**
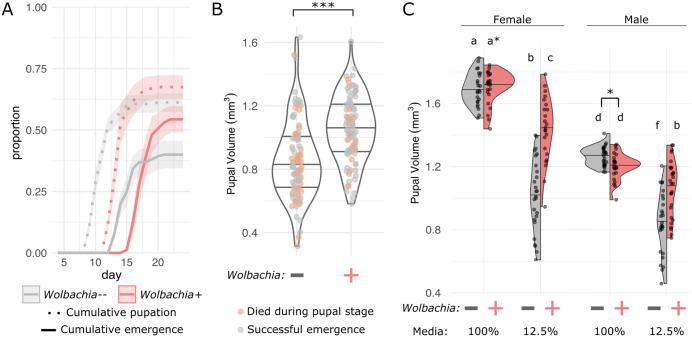
*Wolbachia*-infected flies attain larger sizes under severe nutrient deficiency. Wild-type DGRP-320 flies, with and without *Wolbachia*, were reared on different concentrations of media to test the impact of *Wolbachia* infection. **(A)** Flies reared on 12.5% strength media. Biological replicates included 20 larvae per each of 10 vials. Dotted and solid lines indicate cumulative pupation and adult emergence, respectively, with shaded regions defining standard error. *Wolbachia*-infected treatments are in red, and *Wolbachia*-uninfected in grey. **(B)** Pupal volumes of wild-type flies with and without *Wolbachia*, derived from the 12.5% media-reared flies in **(A)**. Orange datapoints indicate pupae that did not eclose into adults. *** p < 0.001. **(C)** Wild-type flies were reared on 100% or 12.5% media and collected during pupal stages P13-P14 [38]. Pupae were sex sorted, measured, and their volume was calculated. Letters indicate statistically significant differences in mean pupal volume between groups (Tukey’s Honest Significant Difference test, p < 0.05). Asterisks indicate significant differences in the underlying distribution of data (for a*, [female, *Wolbachia*+ flies, 100% media], this is a significant non-normal distribution; for b*, [male flies, 100% media], the distributions were significantly different between *Wolbachia* + /- groups).

Network analyses revealed that 418 of the DEGs (28.3%) form two high confidence networks (Figs 1C and B in S1 Text), each of which was also associated with significantly overrepresented STRING clusters (S5 Table). Because the list is long and somewhat redundant due to the nested nature of functional annotations, we summarize the findings as they relate to Fig 1C below. The larger network contains three clusters, each with proteins corresponding to different significantly overrepresented functional categories (Fig 1C, S1,S2 and S3 Tables). The “C-1” cluster is strongly downregulated in *Wolbachia*-infected flies and contains many of the aforementioned unnamed genes. While unnamed, many of these proteins are predicted peptidases. DEGs were significantly overrepresented for terms related to transmembrane transport and protein localization to microtubule, and the related genes are also largely clustered in “C-1”. The “C-2” cluster is primarily upregulated in the presence of *Wolbachia*. Significantly overrepresented terms that cluster in C-2 include signal, serine proteases (e.g., the Jonah proteases, “Jon*”), extracellular, hydrolase, digestion, carboxypeptidase, and transmembrane transport. The “C-3” cluster is also upregulated in the presence of *Wolbachia*. Significantly overrepresented terms here are related to translation, specifically ribosome biogenesis and tRNA modification. There were also many DEGs in this cluster related to purine nucleotide metabolism, including genes such as *prat*, *prat2*, and *ade3*. Finally, the second, smaller network (Figs 2C–4) is strongly upregulated in the presence of *Wolbachia* and made up of proteins that correspond to the significantly overrepresented terms signal, cuticle development, and chitin. Interestingly, this network also includes *ipod*, the interacting partner of Dnmt2 (an RNA methyltransferase previously implicated in *Wolbachia*-mediated pathogen blocking [32]).

### *Wolbachia* is beneficial under nutrient limited conditions

Given the strong impact of *Wolbachia* on larval gene expression, we hypothesized that *Wolbachia* would impact fly developmental trajectories. To test this, we reared wild-type flies (again, DGRP-320) on 100% strength and 25% strength media and quantified pupation and adult emergence (Fig 2A and 2B). We found a significant interaction between *Wolbachia*, media strength, and time that impacted pupation (F_1,1063 _= 24.7147, p < 0.0001) and adult emergence (F_1,1063 _= 22.2897, p < 0.0001). Additionally, we found a significant interaction between the presence of *Wolbachia* and media strength (wild-type pupae: F_1,1063 _= 0.6517, p = 0.0063; wild-type adults: F_1,1063 _= 0.1746, p = 0.0062; *w*^145^ pupae: F_1,711 _= 8.8129, p = 0.0009; *w*^145^ adults: F_1,711 _= 19.0998, p < 0.0001), along with a significant impact of *Wolbachia* infection alone on pupation and adult emergence (wild-type pupae: F_1,1063 _= 0.2874, p < 0.0001; wild-type adults: F_1,1063 _= 0.5375, p < 0.0001; *w*^145^ pupae: F_1,711 _= 10.5483, p < 0.0001; *w*^145^ adults: F_1,711 _= 7.0718, p < 0.0001). For example, the wild-type flies with and without *Wolbachia* reared on 100% strength media reached adulthood in 82% and 81% of cases, but on the 25% strength media these were reduced to 75% and 66%, respectively. The *Wolbachia*-mediated advantage was more prominent for a second fly genetic background, *w*^145^ (Fig 2C and 2D). Even on 100% strength media, the *Wolbachia*-free *w*^145^ flies experienced a 5% reduction in adult emergence relative to *Wolbachia*-infected flies (Fig 2C). When reared on 25% strength media, the *Wolbachia*-infected *w*^145^ flies were more than twice as likely to reach adulthood (70% versus 31%, Fig 2D). Across these assays, we found delays in entering pupation are approximately equal to the delay in adult emergence: *i.e.,* the time spent in metamorphosis did not change as a factor of *Wolbachia* infection (Fig 2). In summary, the *Wolbachia*-mediated advantage was driven by (1) *Wolbachia-*infected flies developing faster as larvae (1–5 days depending on genotype, Fig 2B and 2D) and a (2) a larger proportion of flies reaching adulthood.

### Interactive effects of *Wolbachia*, fly genotype, and diet impact fly metabolism

Considering the complex relationship between *Wolbachia* infection, host genotype, and *Drosophila* development, we decided to further examine these relationships using semi-targeted LC-MS-based metabolomics to measure the relative abundance of 259 metabolites in the same two genetic strains (wild-type DGRP-320, and *w*^145^) raised on either 100% or 25% media (Fig 3; S6,S7 and S8 Tables). We focused on L2 flies to enable a more direct comparison to the RNA-seq data (Fig 1), and the relative ease of staging as compared to later developmental stages. The resulting data revealed that *Wolbachia* induced significant differences in the metabolome of both strains under both dietary conditions (Fig 3A and 3B). Moreover, the associated changes in the fly metabolome largely correlated with the strength of the developmental phenotype. For example, *Wolbachia* had reduced influence on the metabolome of wild-type flies grown on 100% media when compared with uninfected controls (Fig 3C and 3E), with only 11 metabolites exhibiting significant differences on the 100% food as compared to the 25 metabolites that were changed on the 25% media. Similarly, the total number of *Wolbachia-*associated metabolites that reached the cutoff threshold for significance in *w*^145^ was also lower in the 100% media (19 vs 29) (Fig 3D and 3F).

Beyond the total number of metabolites that were changed in *Wolbachia*-infected and uninfected larvae, several of the significantly altered compounds function within metabolic pathways that are clearly associated with *Wolbachia* infection. Specifically, in 3 of 4 analyses, *Wolbachia* infection resulted in reduced levels of the purine degradation products allantoin and allantoate (Fig 3C,3D and 3F), whose production is associated with the antioxidant properties of uric acid [34]. *Wolbachia* infected *w*^145^ flies also displayed decreased accumulation of kynurenic acid and cystine – amino acid derived molecules that are involved in the cellular response against oxidative stress (Fig 3D and 3F) [35–37]. Our analysis also revealed decreases in pantothenate, a precursor to coenzyme A, thus suggesting that *Wolbachia* influences the abundance of a key molecule in mitochondrial metabolism. Finally, we would highlight the interactive impacts on S-adenosyl-L-homocysteine (SAH) abundance in the wild-type flies, an intermediate molecule in the SAM cycle, glutathione biosynthesis, and one carbon metabolism, and thus directly involved in the cellular oxidative stress response and nucleotide biosynthesis.

### *Wolbachia* supports development and larger size under extreme nutrient depravation

We then wanted to explore if these impacts on fly development and metabolism would be further exacerbated under even more challenging nutritional conditions. Given that the effect size for the *Wolbachia* advantage was genotype dependent and that there was a more subtle impact of *Wolbachia* in the wild-type flies, we wondered if an even weaker media would result in a stronger advantage for the *Wolbachia*-infected flies. Conversely, *Wolbachia* could be a burden under more significant nutritional stress. Indeed, when the wild-type flies were raised on 12.5% strength media, we again saw significant effects of the interaction between *Wolbachia* and time on the rate and success of pupation and adult emergence (Fig 4A; pupae: F_1,347 _= 77.135, p < 0.0001; adults: F_1,347 _= 58.9296, p < 0.0001). However, *Wolbachia*-infected flies now developed slower than their uninfected counterparts (~3 days delayed), but they pupated at higher rates (68% versus 61%) and were more likely to reach adulthood (54% versus 40%, Fig 4A). Furthermore, although *Wolbachia*-infected fly development was slower, it resulted in them attaining, on average, a 23% larger size at pupation (F_1,191 _= 32.076, p < 0.0001, Fig 4B). Additionally, *Wolbachia*-infected flies experienced lower levels of pupal mortality (19% versus 35%), perhaps related to the finding that flies that died during pupation were significantly smaller (F_1,191 _= 4.625, p = 0.0328, Fig 4B).

We wondered whether differences in pupal size were influenced by differential impacts of *Wolbachia* on male versus female flies. To determine if sex was a contributing factor to the *Wolbachia*-mediated size difference, we measured sex-sorted wild-type pupae*,* reared on either 100% or 12.5% media, with and without *Wolbachia* (Fig 4C). We found significant effects of sex and its interaction with *Wolbachia* or media on pupal size (sex*Wolbachia: F_1,194_ = 9.365, p = 0.0025; sex*media: F_1,194_ = 17.736, p < 0.0001; sex: F_1,194_ = 265.067, p < 0.0001). As in our previous assay, flies with *Wolbachia* produced significantly larger pupae than those without *Wolbachia* when reared on the 12.5% media (p < 0.0001). Additionally, this size increase with *Wolbachia* infection was more pronounced in females (41% larger) than in males (26% larger). In contrast, there were no significant differences in mean pupal size due to *Wolbachia* for either sex on 100% media (females: p = 0.9999; males: p = 0.8274). However, in these groups, there were significant differences in the distribution of pupal sizes. Specifically, while uninfected female pupae on 100% media followed a normal distribution (Shapiro-Wilk: W = 0.9778, p = 0.8513), the infected female pupal sizes significantly deviated from a normal distribution (Shapiro-Wilk: W = 0.9012, p = 0.0228). Similarly, pupal size distributions were significantly different between the *Wolbachia* infected and uninfected males derived from 100% media (Kolmogorov-Smirnov: D = 0.3896, p-value = 0.0267).

### The *Wolbachia* advantage is not mediated by the gut microbiome

**Fig 5:**
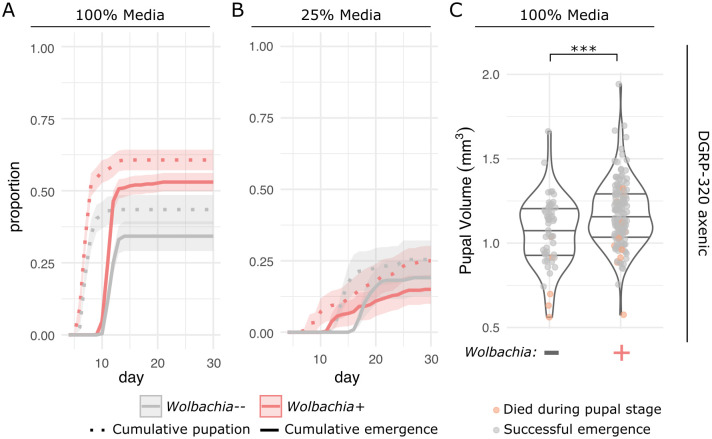
The *Wolbachia* developmental advantages are not mediated by the microbiome. Wild-type DGRP-320 flies, with and without *Wolbachia*, were reared on different concentrations of media under axenic conditions to test the impact of *Wolbachia* infection in the absence of the microbiome. Dotted and solid lines indicate cumulative pupation and adult emergence, respectively, with shaded regions defining standard error. *Wolbachia*-infected treatments are in red, and *Wolbachia*-uninfected in grey. **(A)** Axenically reared flies on 100% and **(B)** 25% strength media. **(C)** Pupal volumes of the axenic-reared flies on 100% strength media derived from **(A)**. Orange datapoints indicate pupae that did not eclose into adults. *** p < 0.001.

Finally, to determine if the developmental advantage was due to direct effects of *Wolbachia* or indirect effects via *Wolbachia*-mediated impacts on the gut microbiome, we performed the developmental assay under axenic conditions (i.e., without a gut or food microbiome). Here, we again focused on the wild-type (DGRP-320) flies, and used 100% and 25% strength media (rather than 12.5%), given that axenic rearing already poses a challenge for the flies [39,40]. Under axenic conditions, we saw a strong interactive effect of *Wolbachia* and time that resulted in significantly more, and faster, pupation and adult emergence for infected flies (Fig 5A; pupae: F_1,1920 _= 153.3796, p < 0.0001; adults: F_1,1920 _= 129.6080, p < 0.0001). Even on the 100% strength media, only 43% of *Wolbachia*-free flies pupated, compared to 61% of *Wolbachia*-infected flies. Again, *Wolbachia*-free flies had higher levels of pupal mortality: 21% of those that pupated did not emerge as adults, as compared to 13% of the *Wolbachia*-infected pupae. While removing the microbiota negatively impacted fly developmental timing as expected, this effect was exacerbated in the *Wolbachia*-free flies (Fig 5A). As compared to the conventionally-reared flies on 100% strength media (Fig 2A), axenic *Wolbachia*-infected flies experienced a two-day developmental delay, and *Wolbachia*-free flies were delayed three days (Fig 5A). The combination of axenic conditions and 25% strength media resulted in especially impaired fly development: less than 20% of flies reached adulthood (Fig 5B). While there were no significant differences in the proportion of flies that pupated or emerged as adults between *Wolbachia*-infected and uninfected flies in these high-mortality conditions (pupae: p = 0.9547; adults: p = 0.6210), it is notable that *Wolbachia*-infected flies on average started emerging five days ahead of the *Wolbachia*-free flies (Fig 5B). Finally, we measured the puparia of the axenic flies reared on 100% strength media and found that *Wolbachia* infection resulted in, on average, 10% larger pupal volumes (F_1,200 _= 11.69, p = 0.0008, Fig 5C), again indicating a significant developmental advantage.

## Discussion

*Wolbachia* has long been appreciated for its diverse impacts on a range of ecdysozoan hosts. Increasingly, there is speculation as to the role *Wolbachia* plays in host metabolism, oftentimes in the context of specific metabolites [12,22,26]. For example, *Wolbachia* contributes to B-vitamin supplementation in a planthopper [19], bed bugs [15], and likely some species of solitary bees [41]. Iron has been the subject of a number of investigations into *Wolbachia*-host interactions [16,17,24,42]. In *Drosophila melanogaster, Wolbachia* infection buffers against stresses associated with low or high concentrations of iron in the diet [24]. *Wolbachia* infection is additionally associated with changes in host ferritin expression and iron homeostasis in other insects such as *Drosophila simulans* and the parasitoid wasp *Asobara tabida* [17]. Given the range of metabolites and physiological processes that have been implicated in *Wolbachia*-host interactions, and the limited understanding of the relationship during the juvenile period, we focused our initial experiments on using a more holistic approach to understanding fly development in the context of diet and *Wolbachia* infection.

Our transcriptomics results contained several surprising findings. First, a huge number of genes were differentially expressed due to *Wolbachia* infection: equivalent to 14% of the active transcriptome. Importantly, these data are derived from the wild-type larvae reared on standard media, conditions in which there is no difference in development rate or success between the *Wolbachia*-infected and -uninfected flies (Fig 2A). We were also surprised to see a clear impact on expression of the immune system. The prevailing hypothesis in the field has been that natively associated *Wolbachia* (e.g., *w*Mel for *Drosophila melanogaster*) do not trigger an immune response, whereas newly initiated infections (e.g., *w*Mel established in *Aedes* mosquitoes) do trigger an immune response [43]. Indeed, while we did not see upregulation of the immune system in *Wolbachia*-infected larvae, we did see strong down-regulation of many immunity-associated loci indicating some level of immune interaction between *Wolbachia* and host in this native association. Other notable findings in the transcriptomes include differential expression of major regulators of metabolism and development such as *Notch*, *InR*, and juvenile hormone signaling related proteins. Genes associated with the digestive system, fat body, muscles, and testes were also significantly more likely to be differentially expressed. While the first three tissues clearly point towards metabolic impacts, we found the pattern of testes-associated genes quite curious given that the analysis focused on L2s which had not been sex sorted. This pattern might reflect sex-specific differences in the timing of gonadal development: ovary morphogenesis does not begin until late L3, whereas testes begin to grow and differentiate substantially during embryogenesis [44]. The earlier development of testes likely results in more power to detect *Wolbachia*-mediated impacts in these tissues as compared to in the developing ovaries, especially when using pooled, homogenized, whole animals. However, it is important to note that these metrics of tissue-biased expression are based on a dataset which is derived from *Wolbachia*-infected flies [30,33,45]. However, even with these caveats, there is clearly a *Wolbachia*-dependent difference in host gene expression across a wide variety of processes.

Given that our gene expression data pointed us towards fly development and metabolism, we sought to test if *Wolbachia* infection manifested in clear organismal impacts. Indeed, we found that *Wolbachia* significantly impacts *Drosophila melanogaster* development, resulting in larger flies that were more likely to reach adulthood. The strength of this phenotype varied by fly genotype and the overall concentration of nutrition in the food: *Wolbachia* infection was highly beneficial under nutrient limited conditions. Importantly, removal of the extracellular microbial community indicated that this advantage was directly mediated by *Wolbachia*. In fact, *Wolbachia* significantly buffered flies against the stresses normally associated with removal of the microbiome [39,40].

Metabolomics analyses of these developing flies identified decreases in purine catabolic products: allantoin, allantoate, and SAH, highlighting metabolic pathways that are associated with *Wolbachia* infection. The larval gene expression data also indicated significantly altered purine metabolism due to *Wolbachia*. And, we previously found an interactive effect of *Wolbachia* infection and the *de novo* purine biosynthesis pathway on virus replication [26], highlighting both the importance of purines to the symbiosis and the complexity of the metabolic landscape. Changes in the concentrations of the purine catabolic product SAH specifically point towards a model linking pathogen blocking with potential roles for *Wolbachia* in host metabolism. The observed changes in SAH are also quite intriguing considering that this molecule is both produced by S-adenosyl-L-methionine (SAM) methyltransferases and also inhibits the activity of these same enzymes [46]. Since *Wolbachia* is proposed to enhance pathogen blocking by interacting with methylation of viral RNA molecules [47], our data suggest that this phenomenon could be the result of nutrient-dependent influences of *Wolbachia* on host metabolism. The relative accumulation of SAH could be indicative of higher levels of methyltransferase activity. Notably, the interacting partner (*ipod*) of the fly’s methyltransferase (*dnmt2)* was strongly upregulated (68-fold) in the *Wolbachia*-infected larvae. *Wolbachia* may mediate effects on the host’s production of SAH, and/or *Wolbachia* might directly produce SAH, as the *Wolbachia* genome encodes for several putative methyltransferases [3]. Indeed, our metabolomics approach does not allow us to identify which organism(s) any of the metabolite pools are derived from. However, given the overlap of several key metabolic pathways [22] we hypothesize that many differences we detect are due to a combination of *Wolbachia*’s own metabolic activity and impacts on the host.

Several other metabolites associated with mitochondrial metabolism and redox balance were significantly altered in *Wolbachia* infected animals. Indeed, *Wolbachia*-mediated changes in redox balance have been the subject of a number of previous investigations [23,48–52], though there are limited functional data to support the ultimate mechanisms responsible. A previously suggested model, based on metabolic differences in adult flies, hypothesized a reprogramming of mitochondrial metabolism, specifically towards non-oxidative metabolism combined with reduced insulin signaling and a hypoxia response [42]. Our transcriptomic and metabolomic data largely support this hypothesis, and indeed, we do see downregulation of the insulin receptor (and upregulation of a negative regulator) in larvae with *Wolbachia*, plus impacts on redox balance.

Our hypothesis that *Wolbachia*’s contributions to metabolism go beyond simple supplementation are further supported by our finding that *Wolbachia* decreases pupal mortality. This was especially notable during extreme nutritional stress (i.e., 12.5% media), where *Wolbachia*-infected flies spent more time in the larval stages, but ultimately were larger and more likely to successfully reach adulthood. These data suggest that *Wolbachia*-free flies enter pupation despite insufficient metabolic reserves, which may mean that *Wolbachia* is delaying the signaling of critical weight, allowing flies to accumulate more metabolic reserves prior to pupation. Indeed, critical weight signaling is directly tied to the insulin signaling-mediated growth of the prothoracic gland [53], and our transcriptomic data indicate that *Wolbachia* infected flies have lower *InR* expression, at least on standard media.

The role of *Wolbachia* as a metabolic symbiont raises numerous questions about the cell biology of *Wolbachia* and how the relationship with the host is regulated. Indeed, the patterns of *Wolbachia* gene expression do seem to be attuned to fly development [31] which could be a strategy for supporting the exponential host growth that occurs during larval stages and mitigating fitness impacts of the infection during reproductive stages. However, it is unknown if *Wolbachia* can specifically export key metabolites via transporters. Or, perhaps flies are “farming” and consuming their *Wolbachia* infections (*e.g.,* via autophagy) and specific metabolites so happen to be abundant in *Wolbachia* cells and also limiting for the fly. As is the case for many symbioses, it is not always clear if a change in the symbiont (*e.g.,* gene expression or titer), is due to the microbe detecting and responding to the host’s status, or the host releasing control of the microbe and “allowing” a change in microbial physiology. *Wolbachia* do encode for a number of proteins that would allow them to detect their environment and regulate gene expression accordingly [54]. However, each *Wolbachia* is enclosed by a host-derived vesicle [55,56], so perhaps the host tightly regulates *Wolbachia*’s “experience” within that enclosed membrane. Changes in *Wolbachia* titer and physiology across development and under different nutritional scenarios likely reflect these evolutionary balancing acts, as is evident by the complex manner by which rapamycin feeding and dietary amino acid supplementation influence *Wolbachia* titers [57].

While beneficial nutritional symbioses in insects are not rare, most of the systems for which this is described are of a much more binary nature. Insects that exclusively feed on unbalanced diets (e.g., blood, plant sap) have obligate microbial symbionts to synthesize metabolites that the host cannot generate *de novo*: the partners complement each other [58,59]. Here, *Wolbachia*’s biosynthetic pathways are largely redundant with the host [22], which perhaps has led us to overlook the relevance of metabolic interactions. Our finding that *Wolbachia* significantly impacts the developmental timing, size, and susceptibility to nutritional stress in a well-studied model such as *Drosophila melanogaster* raises the question of the broader relevance of *Wolbachia* infections to insect metabolism and development.

## Materials and methods

### Fly husbandry

*Drosophila melanogaster* stocks with or without their native *Wolbachia* strain “*w*Mel” were maintained on standard Bloomington cornmeal-agar medium at 25 °C on a 24-hour, 12:12 light:dark cycle under density-controlled conditions and 50% relative humidity. *Wolbachia* colonization status was confirmed with PCR assays using *Wolbachia*-specific 16S primers WspecF and WspecR [60]. Genotypes used in nutritional assays (below) included: DGRP-320 (RRID:BDSC_29654), a *Wolbachia*-infected isogenic wild-type strain with genome sequence available which we refer to as “wild-type” [61], and a *Wolbachia*-infected *white* line, *w*^145^ (RRID:BDSC_145). *Wolbachia*-cleared counterpart stocks were generated with antibiotics via three generations of tetracycline treatment (20 μg/mL in the fly food), followed by re-inoculation of the gut microbiome by transfer to bottles that previously harbored male flies from the original stock that had fed and defecated on the media for one week [62]. Stocks were allowed to recover from any potential transgenerational effects of the antibiotic treatment for at least an additional ten generations prior to use in any experiment and re-screened for *Wolbachia* infection immediately prior to all experiments.

### RNA-Seq of larvae

Pools of second instar (L2) DGRP-320 larvae with or without *Wolbachia* were processed for transcriptomic analyses. We specifically selected L2s because (A) this is a mid-point of larval development, and (B) because L2s are a relatively stable instar that can be tightly synchronized and staged [63,64]. For each infection status, three replicate vials were initiated by allowing five pairs of adult flies to mate and lay eggs on standard Bloomington cornmeal-agar medium for 8 hours. After this period, the adults were removed, and larvae were maintained under standard rearing conditions for an additional 60 hours (i.e., until the mid-L2 stage). At this point larvae were removed from vials with a dissecting pin, morphologically verified to be at the L2 stage based on anterior spiracle and tracheal morphology, and transferred to a microcentrifuge tube containing sterile 1X phosphate buffered saline (PBS). From each replicate vial (collected in a randomized order), we generated a pool of 20 L2s which was washed three times with 1X PBS. After washing, the PBS was removed, and larvae were flash frozen in liquid nitrogen. Total RNA was extracted from each pool using the Monarch Total RNA Miniprep Kit (New England Biolabs) including on-column DNase treatment. RNA-seq libraries (n = 6 total, three of each infection status, as described above) were prepared by Novogene Co, Ltd. and followed a standard strand-specific Illumina-compatible preparation (New England Biolabs) including purification of mRNA using oligo-dT beads. Libraries were sequenced on an Illumina NovaSeq platform to generate ~9Gb of paired end 150 bp reads per library. Reads were mapped to extracted reference transcripts of the *Drosophila melanogaster* reference genome (release 6.48) [65] using the RSEM v. 1.3.0 [66] programs ‘rsem-prepare-reference’ and ‘rsem-calculate-expression’, employing the STAR v. 2.3.5a aligner [67]. Transcript abundance was summarized and imported to R v. 4.3.0 [68] with tximport v. 1.28.0 [69] for use in downstream analyses. Genes deemed “not expressed” (<1 count per million in <2 samples) were removed from the dataset prior to subsequent analysis. Differential gene expression was determined with EdgeR v. 3.42.4 [70,71], employing a TMM normalization, dispersion calculation, and a generalized linear model, with quasi-likelihood F-tests (function ‘glmQLFit’). Genes that were significantly differentially expressed were defined as those with a false discovery rate (FDR) q-value of <0.05 and |log2FC| of >0.5, following best practices based on the size of our dataset [72]. Mitochondrial genes in the DEG dataset were extracted along with their annotations in mitoXplorer 2.0 [73,74]. We determined enrichment with PANGEA [33] using all DEGs as the test set, all expressed genes (S2 Table) as the background, and gene set categories of ‘KEGG Pathway D. mel’, ‘Preferred tissue’, and all three *Drosophila* GOslim gene sets (Biological Process, Cellular Component, Molecular Function). Gene sets with p < 0.05 after Benjamini & Hochberg corrections for multiple testing, or >2 fold-change with uncorrected p-value of p < 0.05 are displayed. DEGs were also clustered into an interaction network using STRING v.1.4.2 [75], implemented in Cytoscape v.3.6.0 [76], which simultaneously assesses overrepresentation of STRING clusters. The confidence threshold for network building was set to 0.85 (i.e., highly stringent).

### Nutritional stress experiments

To generate poor media, Nutri-fly Bloomington Formulation (Genesee Scientific 66–121) was prepared according to manufacturer’s instructions and diluted to indicated percentages while maintaining the full concentrations of agar (5.3 g/L) and propionic acid (4.8 ml/L). Five mL of media was aliquoted into each vial. Flies (either DGRP-320 wildtype, or, *w*^145^) were placed in mating cages on grape agar plates streaked with yeast paste and acclimated for 24 hours, prior to initiating 4-hour egg lays. Upon hatching, < 4-hour old L1 larvae were transferred to media vials (n = 20 per vial), and development was scored every 24 hours. Pupation was defined by eversion of the anterior spiracles [77]. Each vial was tracked until all flies eclosed, or until three consecutive days without additional adult emergence.

### Axenic assays

To test if the gut microbiome was playing a role in *Wolbachia-*mediated developmental phenotypes, we reared DGRP-320 wild-type flies with and without *Wolbachia* on 100% and 25% strength media under axenic conditions (i.e., without the gut or food microbiome). 10 mL of media (prepared according to protocols in “nutritional stress experiments”) was aliquoted into polypropylene wide vials, capped with a Cellulose Acetate Flug (Flystuff 49–101), and autoclaved on liquid setting for 20 minutes to sterilize. Embryos were collected on grape agar with yeast paste as described previously and transferred into 70-micron mesh cell strainers (Falcon 352350). Embryos in the cell strainers were washed with *Drosophila* embryo wash solution (7% NaCl, 0.5% Triton X-100) to remove food debris. After an initial wash, embryos were immersed in a 10% bleach solution for 3 minutes, with gentle mixing every 30 seconds. The embryos were then washed with sterile *Drosophila* embryo wash solution to remove excess bleach. Embryos were subjected to a final rinse with sterile 1XPBS and transferred to a sterile agar culture plate (2% agar in deionized water) stained with blue gel food coloring to facilitate contrast for counting. A flame-sterilized probe was used to transfer 20 embryos into each vial of sterile media, and developmental assays were carried out as described above.

### Pupal size measurements

After selected developmental assays in which flies were reared all the way to adulthood, pupal casings and dead pupae were removed from vials using a wet paint brush and transferred to glass slides. For assays in which we needed to sex the measured pupae, we instead allowed five pairs of flies to mate and lay eggs on the appropriate media for four days, after which the adults were cleared, and offspring were allowed to develop under standard rearing conditions. Approximately 50 pupae per condition were collected at pupal stages P13-14 [38], transferred to glass slides, and sexed based on the presence of sex combs prior to measurement. All pupae on glass slides were imaged with brightfield microscopy on an ECHO Revolve at 4X. Images were manually annotated in Echo Labs Pro software to measure sizes. Pupal length was defined as the distance between the base of the posterior spiracles to the midway point between the anterior spiracles. Width measurements were taken at the widest part of the pupa. The volume of each pupa was calculated assuming a prolate spheroid shape [V = (4/3) π (width/2)^2^ (length/2)] (Fig C in S1 Text) [78,79].

### Metabolomics

We reared L2 larvae of both wild-type (DGRP-320) and *w*^145^ flies, with and without *Wolbachia*, on 100% and 25% BDSC media for metabolomics analyses. Larvae were collected and processed for metabolomics following published protocols and best practices [80,81]. For each of the eight conditions (genotype*Wolbachia*media), we prepared a minimum of six replicate vials. The vials were initiated and synchronized L2 larvae were staged and collected following the procedures noted above (see “RNA-Seq of larvae”), with the exception that larvae were transferred to microcentrifuge tubes containing sterile ice-cold 0.9% sodium chloride (NaCl). Approximately 30 larvae originating from the same vial of media were collected and pooled into a single replicate, resulting in a minimum of six independent biological replicates per condition. Larvae were rinsed in 0.9% ice cold NaCl three times, gently pelleted, the liquid was removed, and then the pool of larvae was immediately flash frozen in liquid nitrogen prior to storing at -80 °C for later processing. Each pool was processed in under 20 minutes to limit decay of metabolites. For metabolite extraction, samples were first transferred to tared 2 mL screwcap tubes containing 1.4 mm ceramic beads pre-chilled in liquid nitrogen. The sample mass was recorded, and tubes were immediately placed back in liquid nitrogen. 800 mL of prechilled (-20 °C) 90% methanol containing 2 µg/mL succinic-d4 acid was added to each sample tube and the sample was homogenized in an Omni Beadruptor 24 for 30 seconds at 6.4 m/s. The samples were removed from the homogenizer, incubated at -20ºC for 1 hr, and centrifuged at 20,000 x g for 5 min at 4 ºC. 600 μl of the supernatant was transferred into a new 1.5 mL microcentrifuge tube and dried overnight in a vacuum centrifuge. Ultra High-Pressure Liquid Chromatography - Mass Spectrometry (UHPLC-MS)-based Metabolomics analyses were performed at the University of Colorado Anschutz Medical Campus, as previously described [82]. Briefly, the analytical platform employs a Vanquish UHPLC system (Thermo Fisher Scientific, San Jose, CA, USA) coupled online to a Q Exactive mass spectrometer (Thermo Fisher Scientific, San Jose, CA, USA). The (semi)polar extracts were resolved over a Kinetex C18 column, 2.1 x 150 mm, 1.7 µm particle size (Phenomenex, Torrance, CA, USA) equipped with a guard column (SecurityGuard Ultracartridge – UHPLC C18 for 2.1 mm ID Columns – AJO-8782 – Phenomenex, Torrance, CA, USA) using an aqueous phase (A) of water and 0.1% formic acid and a mobile phase (B) of acetonitrile and 0.1% formic acid for positive ion polarity mode, and an aqueous phase (A) of water:acetonitrile (95:5) with 1 mM ammonium acetate and a mobile phase (B) of acetonitrile:water (95:5) with 1 mM ammonium acetate for negative ion polarity mode. The Q Exactive mass spectrometer (Thermo Fisher Scientific, San Jose, CA, USA) was operated independently in positive or negative ion mode, scanning in Full MS mode (2 μscans) from 60 to 900 m/z at 70,000 resolution, with 4 kV spray voltage, 45 sheath gas, 15 auxiliary gas. Calibration was performed prior to analysis using the Pierce Positive and Negative Ion Calibration Solutions (Thermo Fisher Scientific). Acquired data was converted from raw to mzXML file format using Mass Matrix (Cleveland, OH, USA). Samples were analyzed in randomized order with a technical mixture injected after every 24 samples to qualify instrument performance. Metabolite assignments were performed using accurate intact mass (sub-10 ppm), isotopologue distributions, and retention time/spectral comparison to an in-house standard compound library (MSMLS, IROA Technologies, NJ, USA) using El-MAVEN (Elucidata, San Francisco, CA, USA).

### Statistics and data visualization

Statistics and data visualization were carried out in R version 3.5.0 [68]. Significant differences in development were assessed with generalized linear mixed-effects models (package: ‘lme4’, function ‘glmer’ [83]) including the proportion of flies that reached a given stage (pupa or adult) as a binomial response, *Wolbachia* presence, day of development, media, and the interaction of the three as fixed effects, and vial as a random effect to account for repeated measures.

Significant differences in pupal volume of flies reared on 12.5% media was assessed with a two-way ANOVA (function ‘aov’) including *Wolbachia*, mortality status, and their interaction as fixed effects. Similarity of metabolomics samples were visualized with non-metric multidimensional scaling (NMDS) using the ‘metaMDS’ function from the R ‘vegan’ package [84] and euclidean distances. Sample collection and data processing for the DGRP-320 and *w*^145^ flies were carried out on separate dates so genotypes were analyzed separately. Differential abundance of metabolites was determined with MetaboAnalyst 6.0 [85] and leveraged normalization by sample mass, square root transformation, and pareto scaling followed by default analysis parameters.

## Supporting information

S1 Text (contains Figs A,B,C). (PDF)

S1 Table RNA-seq library statistics. (XLSX)

S2 Table Expression data for all expressed genes. (XLSX)

S3 Table Differentially expressed *Drosophila melanogaster* L2 genes. (XLSX)

S4 Table Statistical results for overrepresented gene sets (PANGEA). (XLSX)

S5 Table Statistical results for overrepresented terms and clusters (STRING). (XLSX)

S6 Table Metabolomics metadata. (XLSX)

S7 Table DGRP-320 metabolite abundance data. (XLSX)

S8 Table *w*145 metabolite abundance data. (XLSX)

S9 Table Development data, Fig 2. (XLSX)

S10 Table Development data, Fig 4A. (XLSX)

S11 Table Pupal size data, Fig 4B. (XLSX)

S12 Table Sexed pupal sizes, Fig 4C. (XLSX)

S13 Table Axenic development data, Fig 5A and 5B. (XLSX)

S14 Table Axenic pupal sizes, Fig 5C. (XLSX)
