# Supplementary material for: The intracellular symbiont Wolbachia alters Drosophila development and metabolism to buffer against nutritional stress
Source: PLoS Genet. 2025 Oct 15;21(10):e1011905. doi: 10.1371/journal.pgen.1011905 (PMC12543281; doi:10.1371/journal.pgen.1011905)
Supplement: S1 Text — (PDF) [file pgen.1011905.s001.pdf]

Supplemental Text S1:

The intracellular symbiont *Wolbachia* alters *Drosophila* development and metabolism to buffer against nutritional stress

Amelia RI Lindsey[1*], Jason M Tennessen[2], Michael A Gelaw[1], Megan W Jones[1], Audrey J Parish[2], Irene LG Newton[2], Travis Nemkov[3], Angelo D'Alessandro[3], Madhulika Rai[2], Nicole Stark[2]

[*]To whom correspondence should be addressed (alindsey@umn.edu)
[1]Department of Entomology, University of Minnesota, St. Paul, Minnesota, USA
[2]Department of Biology, Indiana University, Bloomington, Indiana, USA
[3]Department of Biochemistry and Molecular Genetics, University of Colorado Anschutz Medical Campus, Colorado, USA

SUPPLEMENTAL FIGURES

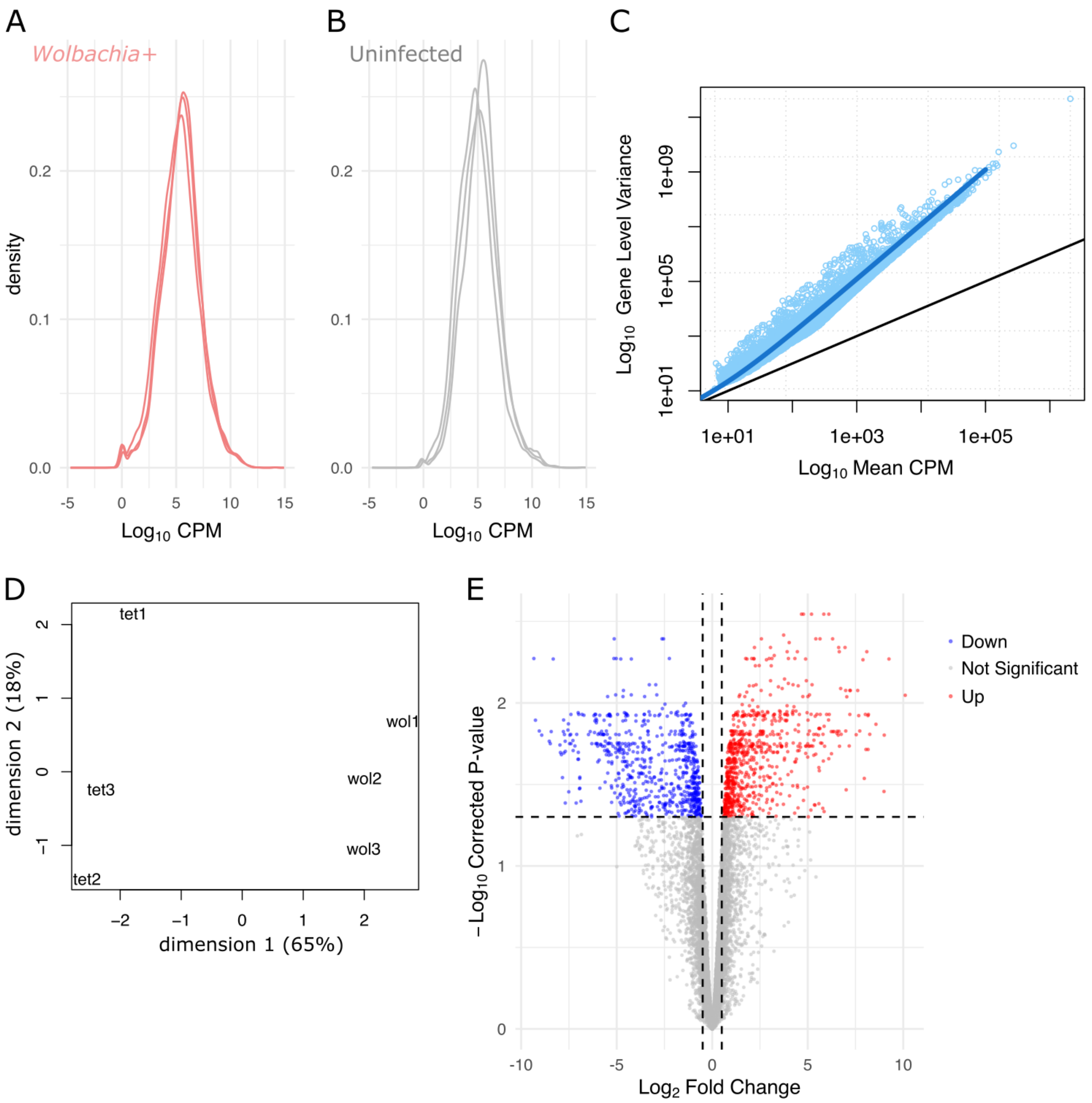

Fig A. Supplemental RNA-seq data. (A) Density plot of normalized gene-counts (CPM) from *Wolbachia*-infected libraries. (B) Density plot of normalized gene-counts (CPM) from *Wolbachia*-uninfected libraries. (C) Mean-variance plot, based on normalized count data (CPM) of all libraries. The black line is the 1:1 correlation, and the blue line is the null hypothesis for a mean-variance relationship for a negative binomial (NB) model with common dispersion. The variance of the data fit well along the NB line. (D) Multidimensional Scaling (MDS) plot of RNA-seq libraries. Generated based on the normalized counts data (CPM). Percentages on each axis indicate the amount of variance explained by the given dimension. (E) Volcano plot indicating differentially expressed genes, where fold change values indicate expression in the *Wolbachia*-infected flies relative to the uninfected. Dotted lines intercepting the log2 fold change axis are set at the |0.5| thresholds. The dotted line intercepting the p-value axis is set at a threshold representing an FDR-corrected p-value of 0.05.

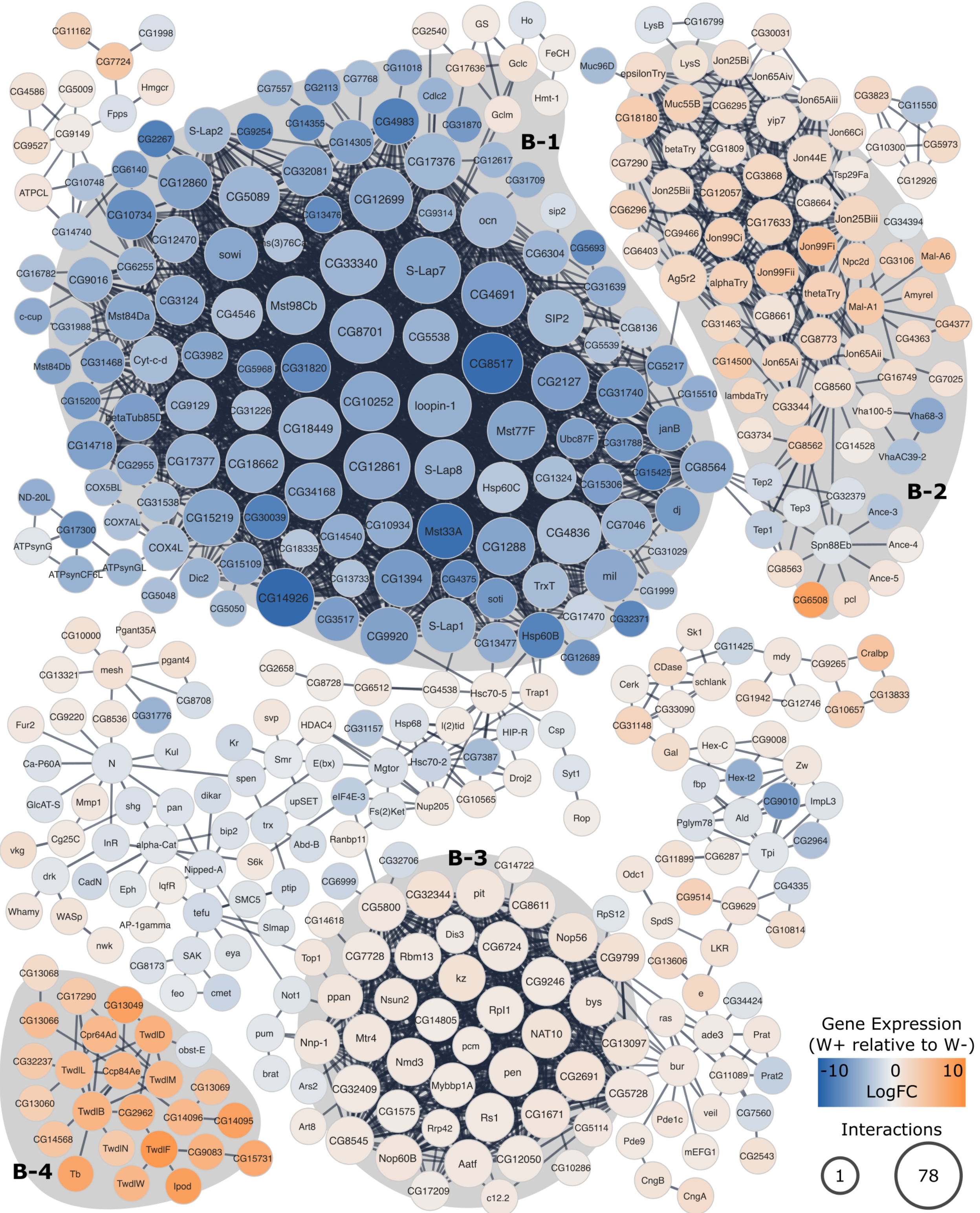

Fig B. Full sized, all-labeled version of RNA-seq network. Core protein-protein interaction networks within the differentially expressed gene set. Nodes are colored according to their change in gene expression relative to uninfected larvae (with orange indicating higher expression in *Wolbachia* infected larvae). The size of each node corresponds to the number of interactions that protein has with other nodes in the network. Shaded regions are clusters of the gene expression

network that are significantly overrepresented with the following functional terms: **(B-1)** uncharacterized peptidases, transmembrane transport, protein localization to microtubule, **(B-2)** signal, serine proteases, extracellular, hydrolase, digestion, carboxypeptidase, transmembrane transport, **(B-3)** ribosome biogenesis and tRNA modification, and **(B-4)** signal, cuticle development, chitin.

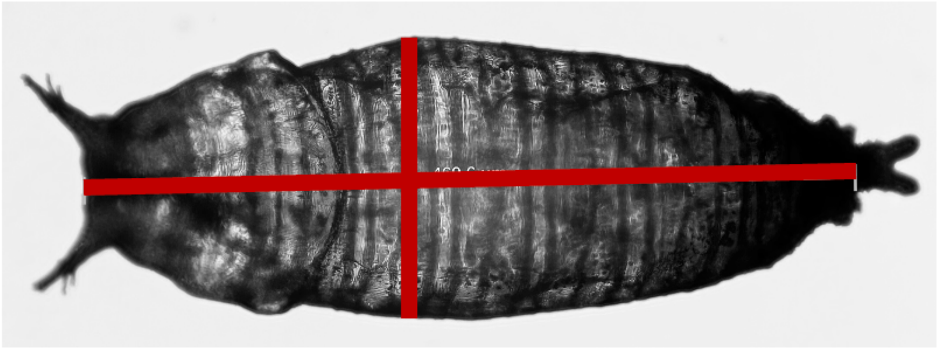

Fig C. Pupal measurements. Pupal volume was calculated based on length and width (red lines) and assuming a prolate spheroid shape [$V = (4/3)\,\pi\,(\text{width}/2)^2\,(\text{length}/2)$].

SUPPLEMENTAL TEXT

Given the large number of differentially expressed genes (DEGs), we looked carefully at the data to ensure there were no obvious false positives that were a result of specific filtering parameters. We identified 10,497 genes that were expressed at >1 CPM in at least two samples (Supplemental Table S1) which were assessed for differential expression. Of these 10,497 genes, 435 genes contained one or more samples with CPM=0. There were no genes for which 4/6 samples had no expression. 47 genes were not expressed (CPM=0) in 3/6 samples: (1) For 7 of these, CPM=0 was recorded for only the tet samples. All 7 were called as DEGs. (2) For 38: CPM=0 in only the *Wolbachia* infected samples and 36 of these were called as DEGs. (3) For 2 genes, it was mixed, and neither of these were called as DEGs. 132 genes were not expressed (CPM=0) in 2/6 samples: (1) For 16 genes these two samples were from different conditions (*Wolbachia* infected versus uninfected). None of these were called as DEGs. (2) For 116 genes, the 2 samples with CPM=0 were from the same condition. 80 of these genes were called as DEGs and in all cases, |log2FC| >4 which is well greater than even especially stringent DEG calls [1]. For all of these genes, the condition in which two samples had CPM=0 had lower expression, with CPM approaching zero in the third replicate. 256 genes had CPM=0 in only one sample. 126 of these genes were called as DEGs and |log2FC| was >3.4 for all of these. To further determine if aberrant low counts were driving DEG calls, we checked for sufficient coverage of DEGs. Specifically, we saw that in the more highly expressed condition (e.g., *Wolbachia*+ for an upregulated gene, or *Wolbachia*— if downregulated), the mean CPM for that condition exceeded 11 (which exceeds the accepted lower threshold of CPM=10). For example, the downregulated gene with the lowest mean CPM in the *Wolbachia* uninfected samples was 11.7. Conversely, the upregulated gene with the lowest mean CPM in the *Wolbachia* infected samples was 13.6. The normalized counts for all expressed genes, for each replicate, are in Supplemental Table S1.

REFERENCES

1. Schurch NJ, Schofield P, Gierliński M, Cole C, Sherstnev A, Singh V, et al. How many biological replicates are needed in an RNA-seq experiment and which differential expression tool should you use? RNA. 2016;22(6):839-51.
